# MRI measurement of the effects of moderate and deep neuromuscular blockade on the abdominal working space during laparoscopic surgery, a clinical study

**DOI:** 10.1186/s12871-023-02201-1

**Published:** 2023-07-14

**Authors:** Piet Krijtenburg, Moira H. D. Bruintjes, Jurgen J. Fütterer, Gert van de Steeg, Frank d’Ancona, Gert Jan Scheffer, Christiaan Keijzer, Michiel C. Warlé

**Affiliations:** 1 Department of Anaesthesiology, Radboudumc, Route 717, Geert Grooteplein Zuid 10, Nijmegen, 6525 GA The Netherlands; 2 Department of Urology, Radboudumc, Nijmegen, the Netherlands; 3 Department of Medical Imaging, Radboudumc, Nijmegen, the Netherlands; 4 3D Lab Radboudumc, Radboudumc, Nijmegen, the Netherlands; 5 Department of Surgery, Radboudumc, Nijmegen, the Netherlands

**Keywords:** Deep neuromuscular blockade, Laparoscopic surgery, Magnetic Resonance Imaging, Surgical conditions

## Abstract

**Background:**

Conflicting data exist regarding the effects of deep neuromuscular blockade (NMB) on abdominal dimensions during laparoscopic procedures.

We performed a clinical study to establish the influence of moderate and deep neuromuscular blockade (NMB) on the abdominal working space, measured by Magnetic Resonance Imaging (MRI), during laparoscopic donor nephrectomy with standard pressure (12 mmHg) pneumoperitoneum under sevoflurane anaesthesia.

**Methods:**

Ten patients were intraoperatively scanned three times in the lateral decubitus position, with pneumoperitoneum maintained by a mobile insufflator. The first scan without NMB (T1) was followed by scans with moderate (T2) and deep NMB (T3). The skin-sacral promontory (S-SP) distance was measured, and 3D pneumoperitoneum volumes were reconstructed.

**Results:**

The mean difference in the S-SP distance was -0.32 cm between T2 and T3 (95% CI -1.06 - 0.42 cm; *p* = 0.344) and + 2.1 cm between T1 and T2 (95% CI 0.81 - 3.39 cm; *p* = 0.006). The mean differences in pneumoperitoneum volume were 166 mL between T2 and T3 (95% CI, 5 - 327 mL; *p* = 0.044) and 108 mL between T1 and T2 (95% CI, -273 - 488 mL; *p* = 0.525). The pneumoperitoneum volume showed high inter-individual variability and no increase in three patients with a high volume at T1.

**Conclusions:**

During laparoscopic surgery in the lateral decubitus position with standard pressure under sevoflurane anaesthesia, deep NMB did not increase the S-SP distance compared to moderate NMB. Moderate NMB increased the S-SP distance by a mean of 2.1 cm (15.2%) compared with no NMB. The mean pneumoperitoneum volume increased slightly from moderate to deep NMB, with high inter-individual variability.

**Trial registration:**

Clinicaltrials.gov ID: NCT03287388.

**Supplementary Information:**

The online version contains supplementary material available at 10.1186/s12871-023-02201-1.

## Introduction

Laparoscopic donor nephrectomy (LDN) is considered the standard technique for live kidney donation [1, 2]. LDN has been found to be associated with reduced analgesic consumption, shorter hospital stay, and faster return to normal physical functioning than the open technique. Recovery after surgery could potentially be improved using low-pressure pneumoperitoneum facilitated by deep neuromuscular blockade (NMB). A meta-analysis performed by our research group showed an improvement in surgical conditions, rated by the surgeons on a Likert scale from 1 to 5, during laparoscopic surgery with deep NMB compared to moderate NMB [3]. However, conflicting data exist regarding the effects of deep NMB on abdominal dimensions during laparoscopic procedures. Vlot et al. [4, 5] found no significant effect of NMB on laparoscopic abdominal dimensions during pneumoperitoneum with computed tomography (CT) measurements in a porcine model. Three human studies [6–8] showed an increase in the skin-sacral promontory (S-SP) distance as a marker of changes in pneumoperitoneum volume. However, no imaging techniques were used in these studies, and the S-SP distance was derived by marking and measuring a laparoscopy instrument.

We performed a clinical study in patients scheduled for LDN to provide more objective data on the influence of deep NMB compared to moderate NMB on pneumoperitoneum volume. We expected to find an increase in pneumoperitoneum volume with deep NMB versus moderate NMB. For this study, we measured the effects of NMB in 1D (S-SP distance) and 3D (pneumoperitoneum volume) using magnetic resonance imaging (MRI).

## Methods

### Ethics

Ethical approval for this study (Ethical Committee N° 2017–3691) was provided by the Medical Research Ethical Committee Oost-Nederland, Philips van Leydenlaan 25, Nijmegen, Netherlands (Vice-Chairperson Dr. J. Roukema) on the 27^th^ of march 2018. Written informed consent was obtained from all participants.

#### Trial registration

Clinicaltrials.gov ID: NCT03287388. September, 2017.

### Patients

#### Study population

Adult patients scheduled for laparoscopic donor nephrectomy were eligible for this study, of which ten were included. We aimed to distribute the included patients by sex as much as possible; however, no patients were excluded based on sex. Exclusion criteria included BMI > 30 kg m^−2^ (to ensure that the patient would fit in the MRI scanner because of the additional space needed for the trocar and drapes), ASA 3 or higher, pregnancy, neuromuscular disease, a contraindication for MRI, known allergy to neuromuscular blocking agents or sugammadex, and severe renal impairment.

### Study method

**Fig. 1:**
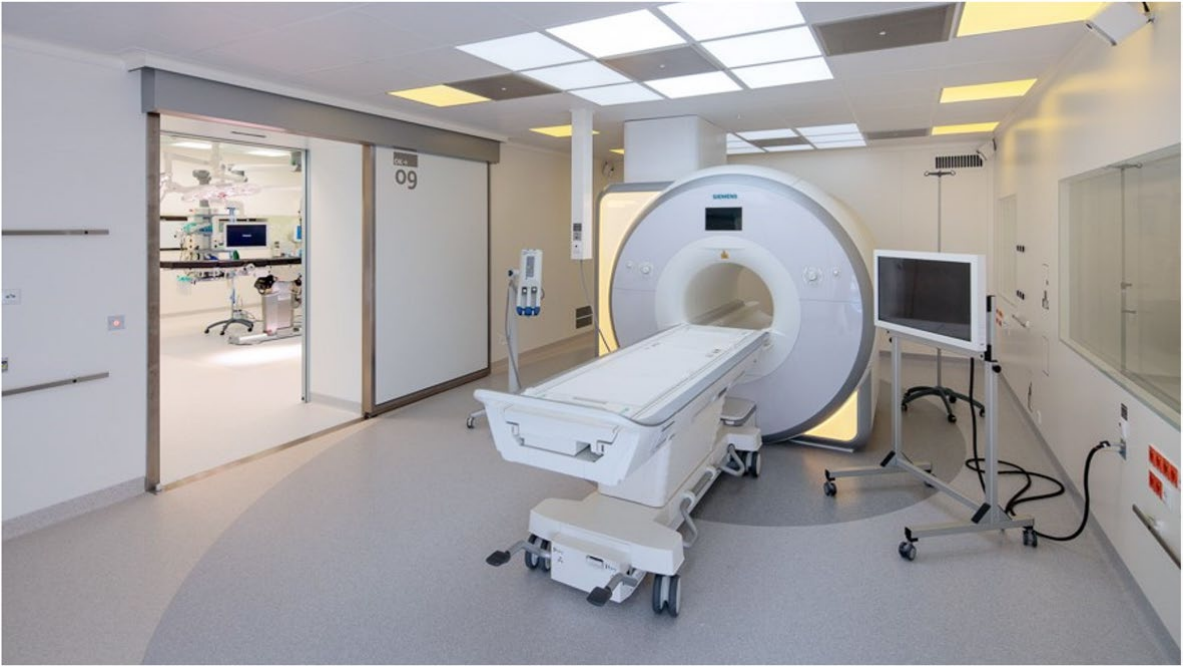
MRI scanner with dockable table and adjacent OR

In the Radboud University Medical Centre in Nijmegen, the Netherlands, a 3 Tesla MRI scanner (MAGNETOM Skyra, Siemens Healthineers, Erlangen, Germany) is present in the operating theatre complex (Fig. 1). The MRI table can be docked directly to the operating table in the adjacent operating room (OR). The patient can be easily slid between the OR and MRI table. This allows for efficient, safe, and rapid intraoperative scanning without an extended transport time or the need for manual transfer to an intermediate MRI-compatible trolley.

All patients had the same attending anaesthesiologist to ensure constant and comparable conditions. After induction of general anaesthesia with propofol and sufentanil (0,3 mcg kg^−1^), the TOF-Watch® SX (Organon, Oss, The Netherlands) was calibrated for acceleromyography at the adductor pollicis muscle with use of the Hand Adapter for optimal preload and stable measurements. Low-dose mivacurium (0,15 mg kg^−1^) was administered to facilitate tracheal intubation. Adequate depth of anaesthesia was maintained with sevoflurane (0.9–1.0 MAC), and end-tidal sevoflurane concentration was monitored both in the OR and MRI scanner to assure comparable conditions in each patient. Pressure-regulated volume-controlled ventilation with 5 cmH_2_O positive end-expiratory pressure and tidal volumes between 6 and 8 ml kg^−1^ were used. Minute ventilation was adjusted to maintain an end-tidal carbon dioxide partial pressure between 31 and 43 mmHg. Additional doses of sufentanil 0,1 mcg kg^−1^ were administered as needed to prevent coughing, straining, or spontaneous breathing from interfering with mechanical ventilation, pneumoperitoneum volume, and scan quality. A nasogastric tube was placed for gastric decompression and removed before the end of surgery. The patient was moved from the supine to the lateral decubitus position. No further repositioning was required for the MRI scan or surgery. In the lateral decubitus position, an open supra-umbilical introduction of the camera trocar was performed, and pneumoperitoneum with a pressure of 12 mmHg was created slowly to avoid pre-stretching. The surgical field and trocar were covered with a sterile drape and the fixed sterile drapes were carefully folded inward. Only the insufflation tube, which was closed with an MRI-compatible Kocher clamp, exited the folded drapings. Because of the use of low-dose mivacurium in all cases, NMB had completely recovered to a Train of Four (TOF) ratio ≥ 1.0 at this point.

The patient was subsequently transported to an adjacent room on the operating table and docked directly into the MRI scanner. The insufflation tube was connected through a hole in the wall to a mobile insufflator (KARL STORZ Electronic Laparoflator Model 26,430,020) in the MRI observation unit. With the mobile insufflator, pneumoperitoneum with a pressure of 12 mmHg was maintained during the scans. At this point, the first scan (T1, no NMB, TOF ratio ≥ 1.0) of the abdomen in the lateral decubitus position, using axial slices at 5 mm intervals (for speed), was made in apnoea by pausing mechanical ventilation. After the first scan, we slid the patient as far as possible from the MRI scanner. This allowed us to be far enough away from the magnetic field to reconnect the TOF-Watch® SX. The electrodes and Hand Adapter were MRI-compatible and still in place to ensure comparable measurements. Rocuronium was slowly titrated to moderate NMB (TOF count 1–3). The mean dose needed was 19,7 mg or 0,26 mg kg^−1^. The TOF-Watch® SX was disconnected when moderate NMB was achieved, and a second scan was performed. As the patient was still docked to the already configured MRI scanner for the first scan, the second scan (T2, moderate NMB, TOF count 1–3) could be performed immediately. After completion of the second scan, an additional high dose of rocuronium (1.2 mg kg^−1^) was administered to ensure deep NMB (Post Tetanic Count (PTC) 0–1). After 3 min the third and final scan (T3, deep NMB, PTC 0–1) was performed. Following the third scan, the patient was transported back to the OR. The level of NMB was assessed to confirm the deep NMB during the final scan. The sterile drapings were carefully renewed, and the laparoscopic procedure proceeded in the lateral decubitus position, deep NMB (titrated towards PTC 0–1) and standard-pressure pneumoperitoneum (12 mmHg). After closure of the fascia at the end of surgery, NMB was reversed with 4 mg kg^−1^ sugammadex. No perioperative adverse events occurred in any of the 10 patients.

**Fig. 2:**
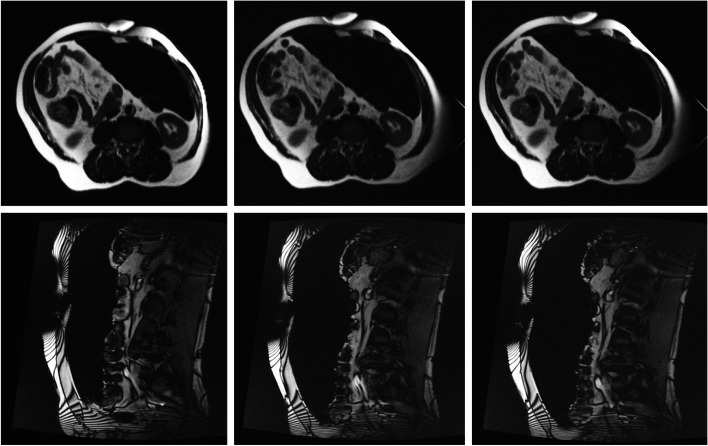
Axial and sagittal MRI slides at different levels of NMB (patient 10). From left to right: T1 (no NMB), T2 (moderate NMB), T3 (deep NMB)

**Fig. 3:**
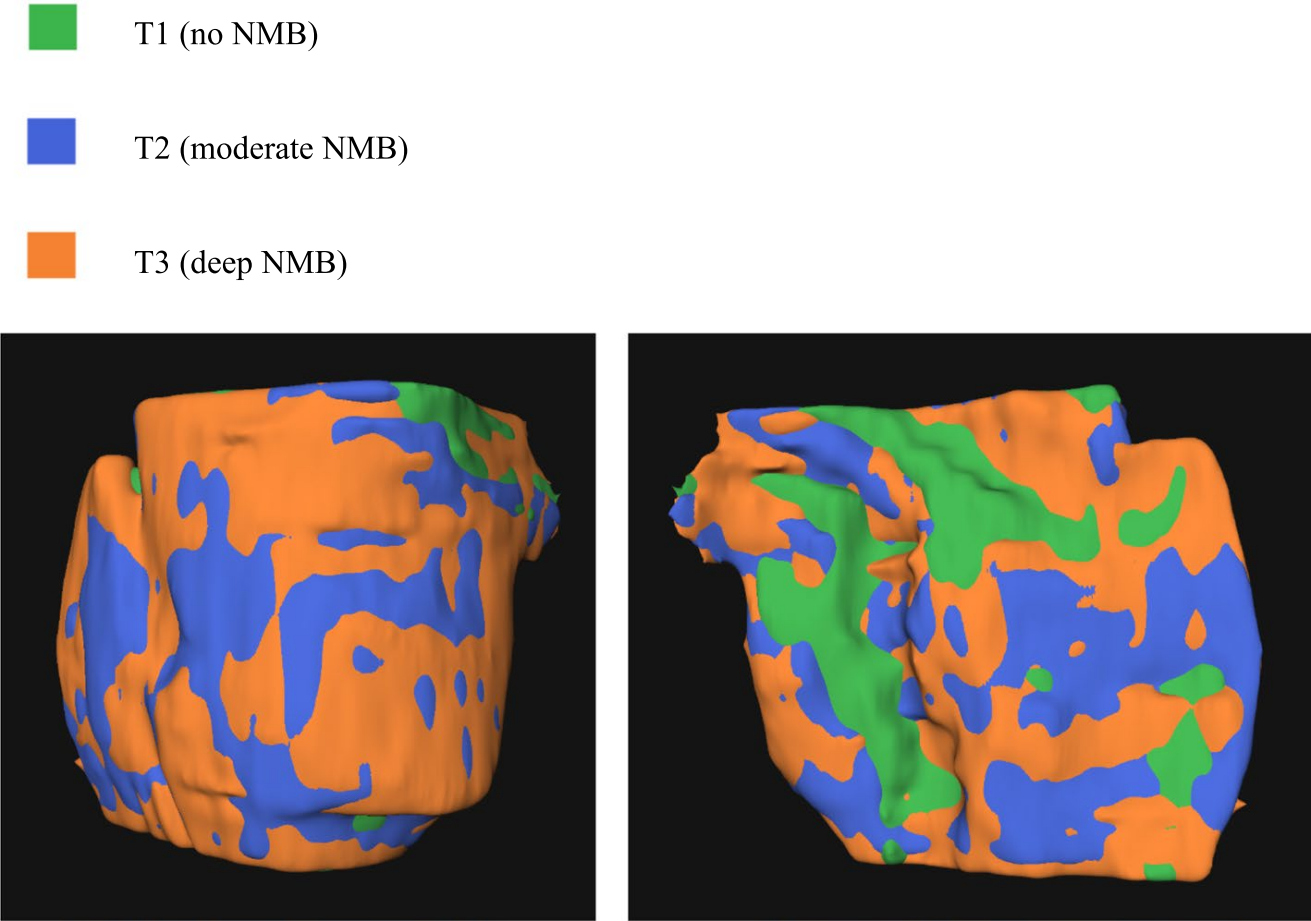
Overlapping 3D reconstructions of pneumoperitoneum shape in patient 10. In each slide of the MRI scans the insufflated area within the peritoneum was manually marked. When combined, a 3D shape could be reconstructed per scan. By overlapping the three 3D reconstructions of the pneumoperitoneum shape the direction of the volume changes could be visualised. Left image: ventral view. Right image: dorsal view

The distance between the skin (supra umbilical trocar entry point) and sacral promontory was measured by a senior attending radiologist who was blinded to the order of the three scans per patient. For the volume measurements on each slide of the MRI scans, the pneumoperitoneum was manually marked. This was done separately by multiple technical medicine students to minimize inter-rater reliability variability and was supervised by a senior attending radiologist and a senior 3D technician. When all slides were combined, a 3D model and volume were reconstructed (with *3DMedX®, v1.2.17.0, 3D Lab Radboudumc, Nijmegen*, and *3D Slicer v4.11.20210226, *www.slicer.org). See Figs. 2 and 3.

### Sample size calculation, study endpoints and statistical analysis

The main study endpoint was a change in the abdominal working space between moderate and deep NMB during standard pressure pneumoperitoneum (12 mmHg), quantified by MRI measurement of the S-SP distance. Based on our previous studies and meta-analysis, we considered a gain of 10 percent in surgical space as a minimal clinically important difference, conforming to a 0.5 difference on a 5-point Likert scale (Leiden-surgical rating scale). Based on the findings of earlier studies measuring S-SP distance [6–8] we calculated the necessary sample size for a paired T-test of at least ten patients to provide 80% power to detect a 0.6 cm difference with a SE of 0.6 cm between moderate and deep NMB (alpha 5%), representing an expected gain of approximately 10% in abdominal working space. Secondary endpoints were the changes in S-SP distance between no NMB and moderate NMB, and changes in pneumoperitoneum volume and 3D shape of the abdominal wall and cavity between no, moderate, and deep NMB. Normal distributed outcome data will be presented as means with 95% CI and skewed data will be presented as median with range.

Statistical analyses were performed using IBM SPSS Statistics Release 25.0.0.1. The Shapiro–Wilk test of normality showed a high probability of a normal distribution for both the S-SP distance and pneumoperitoneum volumes; therefore, a paired samples T-test was used and data were presented as means with 95% CI.

## Results

Eighteen patients were screened for inclusion, eight patients were excluded based on predefined criteria (*n* = 3) or lack of informed consent (*n* = 5). Ten patients were included in the study, after obtaining written informed consent, from June 2019 to December 2020. See Table 1.

**Table 1:** Patient characteristics

Patient	Sex	Age (y)	Height (cm)	Weight (kg)	BMI	Pregnancies	Previous abdominal surgery (Y/N)
1	M	64	176	90	29.1	-	N
2	M	58	184	88	26.0	-	N
3	F	48	174	64	21.1	1	N
4	F	57	169	83	29.1	6	Y
5	F	51	163	62	23.3	3	Y
6	M	53	179	89	27.8	-	N
7	F	69	167	74	26.5	2	Y
8	F	57	171	65	22.2	0	N
9	F	54	167	75	26.9	2	N
10	M	55	173	84	28.1	-	N

The mean difference in the S-SP distance (Table 2) was -0.32 cm between T2 and T3 (2.0% decrease from T2; 95% CI -1.06 to 0.42 cm; *p* = 0.344). This difference was +2.1 cm between T1 and T2 (15.2% increase from T1; 95% CI 0.81 to 3.39 cm; *p* = 0.006) and +1.78 cm between T1 and T3 (12.9% increase from T1; 95% CI 0.67 to 2.88 cm; *p* = 0.006), respectively. Large differences were observed in the S-SP distances measured without NMB at T1. Male patients had higher baseline S-SP distance than female patients: mean 16.6 cm (95% CI 13.8 to 19.4 cm) vs 11.5 cm (95% CI 10.4 to 12.5 cm), respectively. Previous abdominal surgery or pregnancy did not seem to affect S-SP distance.

**Table 2:** MRI measurements of skin – sacral promontory distance

Patient	T1 (cm)	T2 (cm)	T3 (cm)
**1**	18.1	19.3	17.7
**2**	18.1	18.6	18.9
**3**^a^	-	-	-
**4**	11.1	13.0	13.4
**5**	11.4	12.6	11.8
**6**	14.6	17.5	18.1
**7**	11.6	17.0	15.1
**8**	12.8	13.1	13.7
**9**	10.4	14.3	13.6
**10**	15.7	17.3	17.5
**Mean (95% CI)**	13.8 (11.5 - 16.1)	15.9 (13.9 - 17.9)	15.5 (13.6 - 17.5)

^a^Excluded from analysis because of technical issue with the mobile insufflator

3D reconstructions of the pneumoperitoneum at each time point were used to calculate volume. Mean differences in pneumoperitoneum volumes (Table 3) were 108 mL between T1 and T2 (3,0% increase from T1; 95% CI, -273 to 488 mL; *p* = 0.525), 166 mL between T2 and T3 (4,5% increase from T2; 95% CI, 5 to 327 mL; *p* = 0.044), and 253 mL between T1 and T3 (7,0% increase from T1; 95% CI, -51 to 557 mL; *p* = 0.090), respectively.

**Table 3:** MRI measurements of pneumoperitoneum volume

Patient	T1 (mL)	T2 (mL)	T3 (mL)
**1**	4982	4736	4876
**2**	5921	5301	5953
**3**^a^	-	-	-
**4**	4379	4191	4303
**5**	2896	3126	3140
**6**^b^	-	2721	3055
**7**	2544	3379	3531
**8**	3047	3154	3192
**9**^b^	2730	2989	2977
**10**	2176	2661	2726
**Mean (95% CI)**	3584 (2463—4706)	3692 (2906—4478)	3837 (2903—4772)

^a^Excluded from analysis because of a technical issue with the mobile insufflator

^b^A technical issue with the MRI scanner where the scanned area was (slightly) shifted between scans. See Discussion

The baseline (T1) volume with no NMB was remarkably larger in three patients (1, 2, and 4). No clear effect of moderate or deep NMB was observed in these patients. A substantial mean increase of 383 mL (14.3% increase) from no NMB to moderate NMB was observed in the other patients.

The 3D reconstructions of T1,T2, and T3 were merged into one image per patient to illustrate where the changes in shape were most pronounced (Fig. 3). In patients with an increasing volume, the expansion was visible in all directions, but slightly more in the ventrolateral direction towards the insufflation-trocar, as might be expected in the lateral decubitus position. This is in concordance with the relatively greater increase in 1D (S-SP distance) compared with the increase in 3D (pneumoperitoneum volume) with NMB.

## Discussion

During laparoscopic surgery in the lateral decubitus position with standard pressure under sevoflurane anaesthesia, deep NMB does not increase the S-SP distance compared with moderate NMB. In all patients, the S-SP distance increased between no NMB and a moderate NMB. Our data are in concordance with those of previous studies. Lindekaer et al. [8] compared no NMB with deep NMB at a pneumoperitoneum pressure of 12 mmHg and found a mean difference of approximately + 1.5 cm and we found a difference of + 1.78 cm. Madsen et al. [7] found a slightly smaller difference of + 0.33 cm (95% CI 0.07 to 0.59; *p* = 0.01). Barrio et al. [6] compared moderate NMB with deep NMB at a pneumoperitoneum pressure of 12 mmHg and found a small difference of + 0.46 cm (95% CI 0.26 to 0.65 cm). We found no significant difference, but our 95% CI’s (-1.06 to 0.42 cm) overlapped with their data.

The mean pneumoperitoneum volume did not increase between no NMB and moderate NMB. Between moderate NMB and deep NMB, there was a small (mean 166 mL) but statistically significant increase. In this dataset, we noticed that in patients with a relatively large pneumoperitoneum volume of > 4000 mL without NMB (patients 1, 2, and 4), there seemed to be no additional effect of NMB. Barrio et al. [6] also found high inter-individual variability in the increase in S-SP distance and insufflated CO_2_ volume, and an increase was not observed in all their patients. Our data suggest that in patients with a lower abdominal volume without NMB (T1), the increase between no NMB and moderate NMB could be substantially larger. This observation supports and is in concordance with the current clinical practice of administering additional neuromuscular blocking agents in patients in whom surgical exposure is suboptimal.

Vlot et al. [4, 5] used a porcine laparoscopy model to investigate the influence of NMB on the abdominal working space, measured by Computed Tomography (CT). They found no significant effect of NMB on the laparoscopic working space and abdominal dimensions. They stated that the results found in earlier studies could be confounded by pre-stretching of the abdominal wall. However, one might question whether the results can be directly translated to clinical practice, because the effects of pneumoperitoneum and NMB might differ between pigs and humans.

An increase in abdominal compliance while maintaining the a pneumoperitoneum pressure of 15 mmHg during long laparoscopic procedures has been observed in a study by Verbeke et al. [9] However, the lower pressure used in this study and the fact that the MRI scans were performed shortly after the start of the pneumoperitoneum will probably have attenuated this effect.

We observed no increase between moderate and deep NMB in S-SP distance, and only a small increase in pneumoperitoneum volume. This could be explained by NMB potentiation [10, 11] by the use of an inhalational anesthetic (sevoflurane). This possible explanation is in concordance with the study of Honing et al., who found that deep NMB does not improve surgical conditions in patients receiving sevoflurane anaesthesia for laparoscopic renal surgery [12]. When using propofol anaesthesia there may be a larger influence of deep NMB on pneumoperitoneum dimensions and this should be addressed in future studies.

The scans were performed with a trocar in situ and a maintained pneumoperitoneum, but without intraoperative surgical stimuli with changing intensity. It is possible that these surgical stimuli would alter abdominal wall muscle tone and contractions, which could result in more pronounced effects of (deep) NMB on surgical conditions during actual laparoscopic surgery. This was observed in an earlier meta-analysis of surgical conditions rated by surgeons themselves during laparoscopic surgery with deep NMB [3] and in a study by Bruintjes et al. [13] showing less intraoperative muscle contractions with deep NMB. Our data suggest that an increase in working space was probably not a significant factor in this positive effect on surgical conditions of deep NMB compared to moderate NBM in studies using a standard pneumoperitoneum pressure of 12 mmHg. When using a low-pressure pneumoperitoneum, the effect of deep NMB on working space may be different, which should be addressed in future studies.

Furthermore, although rocuronium was carefully and gradually titrated towards moderate NMB, some patients could have reached a slightly deeper NMB at the time of the actual MRI scan at T2 because of a delayed peak effect. However, because of the relative low dose of rocuronium used to reach moderate NMB (mean 19.7 mg or 0.26 mg kg^−1^) it is very unlikely any patients reached deep NMB [14]. Unfortunately, it was technically impossible to continually measure NMT in the MRI scanner to verify the level of NMB at the exact time of the MRI scan.

### Strengths and limitations

The strengths of this study are the unique design with an MRI scan during surgery with objective measurements compared with subjective ratings of surgeon satisfaction during surgery. However, because of the unique setup this study is hard to repeat and comprises a relatively small number of patients and consequently a small dataset.

Because of the complex logistics involved in this study, we encountered some technical difficulties. To minimize the risk and the additional time the patient had to be under general anaesthesia, we had to make some concessions in scan quality. We scanned the patient in the lateral decubitus position instead of the supine position to avoid the risks associated with repositioning the patient with a trocar in situ. Because of the slow scan speed of the MRI scanner, we performed three quick scans with a relatively low resolution and large 5 mm intervals between axial slices for volume measurement of the pneumoperitoneum. To measure the S-SP distance, a higher resolution with shorter intervals was used because only a small part of the abdomen needed to be scanned. Because the total pneumoperitoneum can be quite large, in most patients, the complete pneumoperitoneum did not fit in a single scan. Therefore, the measured volumes should not be interpreted as total pneumoperitoneum volume. Performing multiple scans per phase of muscle relaxation would have been both impractical and time-consuming. For reliable comparison of the volume between the three scans in each patient, the same number of axial slices was used per scan for each analysis.

In patients 6 and 9, there was a (slight) mismatch in the scanned area between scans. For an unclear reason, the scanned area was shifted after the patients were slid in and out of the MRI between scans. This did not affect the primary endpoint (S-SP distance) but it did affect the volume measurements. In patient 9, the scans were only slightly shifted, and only adequately overlapping parts were used for analysis. In patient 6, the first scan had too little overlap with the other scans to be useful. We did not reach the intended sample size from our sample size calculation (9 patients instead of 10) because patient 3 was excluded from analysis after a technical issue with the mobile insufflator. Therefore these results could be underpowered and so they must be interpreted from a physiologic-descriptive point of view. We intended to include 5 male en 5 female patients. We ended up with 4 male and 5 female patients because patient 3 was excluded from analysis.

Manual analysis of the scans to identify the pneumoperitoneum in each slide was sometimes difficult because of the relatively low resolution and artifacts caused by the trocar.

## Conclusion

During laparoscopic surgery in the lateral decubitus position with standard pressure under sevoflurane anaesthesia, deep NMB does not increase the S-SP distance compared with moderate NMB. Moderate NMB increased the S-SP distance by a mean of + 2.1 cm (15.2% increase) compared to no NMB. The mean pneumoperitoneum volume increased slightly from moderate to deep NMB. Our data suggest that an increase in working space was probably not a significant factor in the positive effect on surgical conditions rated by surgeons of deep NMB [3] compared to moderate NMB reported in earlier studies using a pneumoperitoneum pressure of 12 mmHg. When using a low-pressure pneumoperitoneum, the effect of deep NMB on working space may be different. We observed high interindividual variability with a greater increase in pneumoperitoneum volume with moderate NMB in patients with a lower initial volume without NMB.

## Supplementary Information

**Additional file 1. **CONSORT 2010 Flow Diagram.

## Data Availability

The datasets used and/or analyzed during the current study are available from the corresponding author upon reasonable request.

## Abbreviations

NMB: Neuromuscular blockade
MRI: Magnetic Resonance Imaging
S-SP: Skin-sacral promontory (distance)
LDN: Laparoscopic donor nephrectomy
OR: Operating Room
PTC: Post Tetanic Count
TOF: Train of Four
ASA: American Society of Anesthesiologists
BMI: Body Mass Index
