# Supplementary figures and images for: MRI measurement of the effects of moderate and deep neuromuscular blockade on the abdominal working space during laparoscopic surgery, a clinical study
Source: BMC Anesthesiol. 2023 Jul 14;23:238. doi: 10.1186/s12871-023-02201-1 (PMC10347813; doi:10.1186/s12871-023-02201-1)

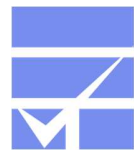

# CONSORT

TRANSPARENT REPORTING of TRIALS

## CONSORT 2010 Flow Diagram

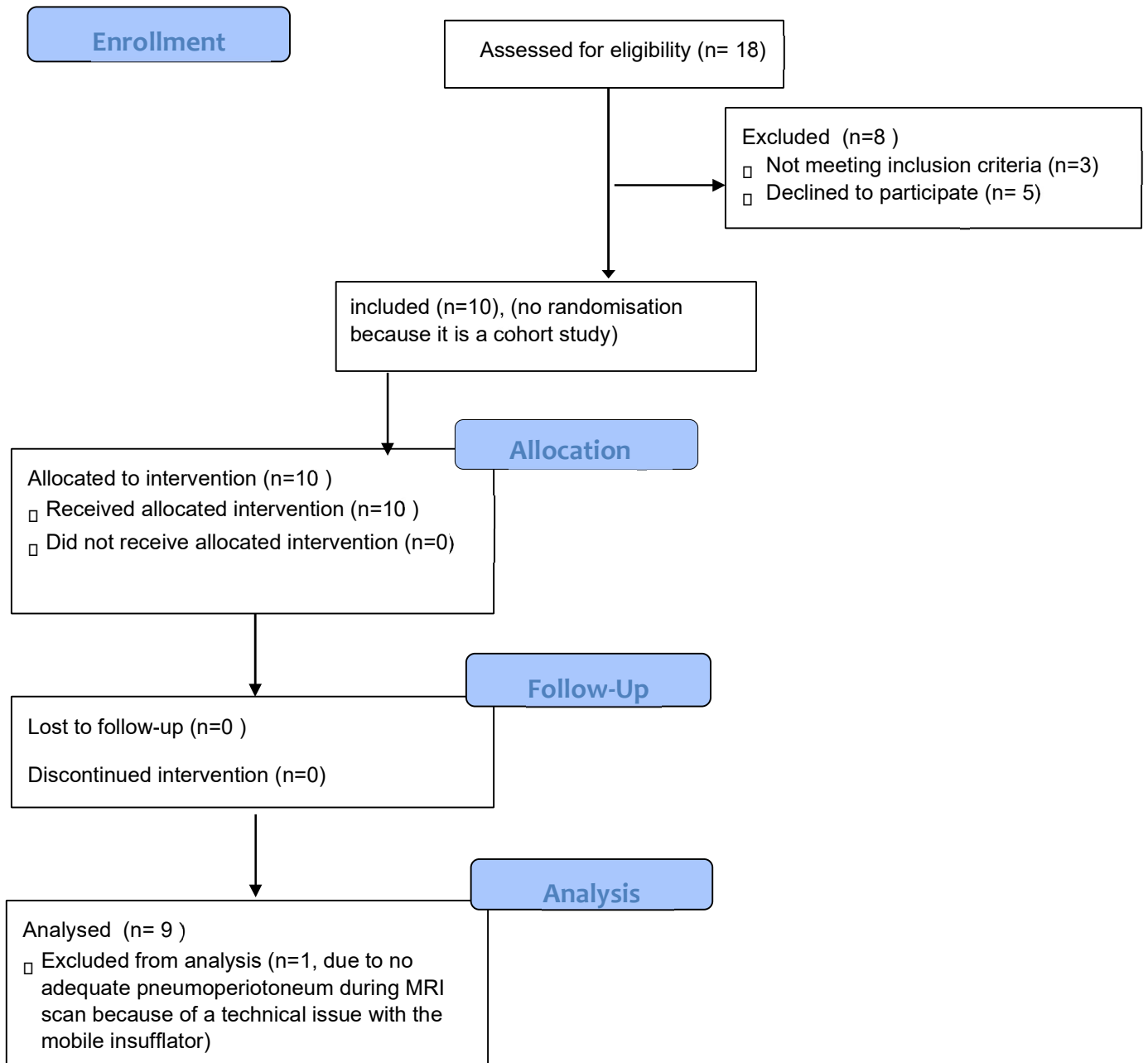

Supplement: Supplementary file 1 — Additional file 1. CONSORT 2010 Flow Diagram. [file 12871_2023_2201_MOESM1_ESM.pdf]
